# Solution NMR Studies of *Mycobacterium* *tuberculosis* Proteins for Antibiotic Target Discovery

**DOI:** 10.3390/molecules22091447

**Published:** 2017-08-31

**Authors:** Do-Hee Kim, Sung-Min Kang, Bong-Jin Lee

**Affiliations:** Research Institute of Pharmaceutical Sciences, College of Pharmacy, Seoul National University, Seoul 151-742, Korea; ozmagic15@snu.ac.kr (D.-H.K.); men0528@snu.ac.kr (S.-M.K.)

**Keywords:** *Mycobacterium tuberculosis*, antibiotic target, nuclear magnetic resonance (NMR), biomolecular interaction

## Abstract

Tuberculosis is an infectious disease caused by *Mycobacterium* *tuberculosis*, which triggers severe pulmonary diseases. Recently, multidrug/extensively drug-resistant tuberculosis strains have emerged and continue to threaten global health. Because of the development of drug-resistant tuberculosis, there is an urgent need for novel antibiotics to treat these drug-resistant bacteria. In light of the clinical importance of *M*. *tuberculosis*, 2067 structures of *M*. *tuberculsosis* proteins have been determined. Among them, 52 structures have been solved and studied using solution nuclear magnetic resonance (NMR). The functional details based on structural analysis of *M*. *tuberculosis* using NMR can provide essential biochemical data for the development of novel antibiotic drugs. In this review, we introduce diverse structural and biochemical studies on *M*. *tuberculosis* proteins determined using NMR spectroscopy.

## 1. Introduction

*Mycobacterium tuberculosis* quickly became a global epidemic in the 18th century and continues to threaten global health in the 21st century. Genome comparison of *M*. *tuberculosis* with other bacteria revealed characteristics of both Gram-negative and Gram-positive bacteria. This unique characteristic of *M*. *tuberculosis* provides insights into the pathogenicity of *M*. *tuberculosis* based on phylogenetic analysis [1].

Tuberculosis (TB) is a powerful infectious disease that has been present in humans for more than 15,000 years. TB spreads via the respiratory tract from infected people or the gastrointestinal route via contaminated food and triggers severe pulmonary diseases [2]. TB causes approximately 2 million deaths every year. Furthermore, current pharmaceutical therapies show clear limits in the cure rate [3]. TB control is highly vulnerable to multidrug resistance (MDR)-TB epidemics because of inadequate treatment and increasing resistance. More than 350,000 new cases of MDR-TB occur annually [4]. In addition, extensively drug-resistant tuberculosis strains (XDR-TB), which are resistant to fluoroquinolones and second-line injectables, have been reported and the use of ethionamide as second-line treatment is not very effective [5]. Because of increases in drug-resistant tuberculosis (MDR-TB and XDR-TB), there is an imminent need for new drug candidates with alternative mechanisms of action.

To discover novel antibiotic candidates, new pharmaceutical agents for *M*. *tuberculosis* that can relieve the current danger associated with drug-resistance should be developed. Bacterial genome-sequencing may be useful for antibiotic resistance detection. Genome-sequencing experiments of pathogenic bacteria have revealed much information and provided valuable contributions to disease surveillance [6]. For *M*. *tuberculosis*, the complete genome sequence of the most well-characterized strain H37Rv contains 4,411,429 base pairs. H37Rv contains more than 4000 genes and encodes 3924 proteins. These proteins can be assigned to several large subgroups according to their functions: hypothetical proteins, metabolic pathways including proteins for carbohydrate and lipid metabolism, transcription-regulating proteins, drug resistance-hosting proteins, and immune-related proteins [7]. In one gene cluster of *M*. *tuberculosis*, 122 genes are related to more than three essential metabolic pathways; among them, 55 genes are predicted to be indispensable for survival and the maintenance of *M*. *tuberculosis* [8]. Based on these bioinformatics data, proteins in *M*. *tuberculosis* contain various novel therapeutic targets.

Considering the clinical importance of *M*. *tuberculosis*, the protein data bank (PDB) contains a total of 2067 structures of the proteins alone and in complex with binding partners (chemicals or proteins) from this bacterium. Among these structures, 2011 structures (97.3%) were determined by X-ray crystallography, 52 structures (2.5%) were determined by nucleic magnetic resonance (NMR) spectroscopy, and four structures (0.2%) were determined by electron microscopy.

Despite its size limitations, NMR spectroscopy is a powerful tool for revealing the atomic structure of macromolecules in solution. NMR can provide essential biochemical data for the development of antibiotic drugs with novel mechanisms of action. Therefore, functional details based on structural analysis of *M*. *tuberculosis* using NMR are significant. In this review, we introduce the diverse structural and biochemical studies of *M*. *tuberculosis* proteins based on NMR experiments. Valuable findings based on chemical shift perturbation and ligand-binding studies reveal information regarding biophysical mechanisms and dynamics of target proteins, which can be applied for anti-tuberculosis drug discovery.

## 2. NMR Structures

**Figure 1:**
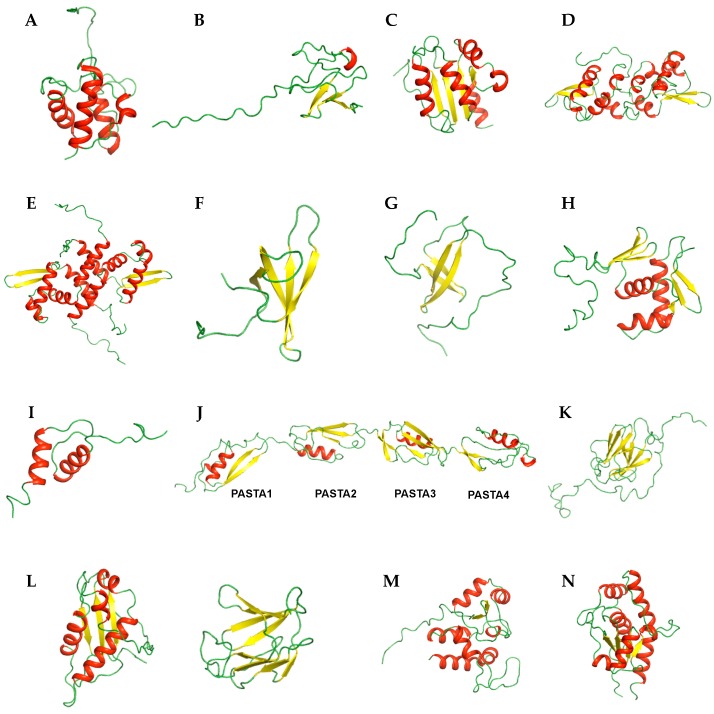
Ribbon representation of NMR structures of *M*. *tuberculosis* proteins. Transport-related proteins (**A**) Rv2244 (PDB ID 1KLP); (**B**) Rv3250c (PDB ID 2KN9); (**C**) Rv1739c (PDB ID 2KLN). Transcription-related proteins (**D**) Rv1994c (PDB ID 2JSC); (**E**) MT3852 (PDB ID 2LKP); (**F**) Rv0639 (PDB ID 2MI6); (**G**) Rv2050 (PDB ID 2M4V). Nucleotide-binding proteins (**H**) J113_05350 (PDB ID 2RV8); (**I**) Rv3597c (PDB ID 2KNG); Ser/Thr Protein kinase-related proteins (**J**) Rv0014c (PDB ID 2KUI); (**K**) Rv1827 (PDB ID 2KFU); (**L**) Rv0020c (PDB ID 2LC0 (Left) and 2LC1 (Right)); (**M**) Rv2175c (PDB ID 2KFS); (**N**) Rv2234 (PDB ID 2LUO). Secondary structural elements, α-helix, β-sheet, and loop are colored in red, yellow, and green, respectively.

**Figure 2:**
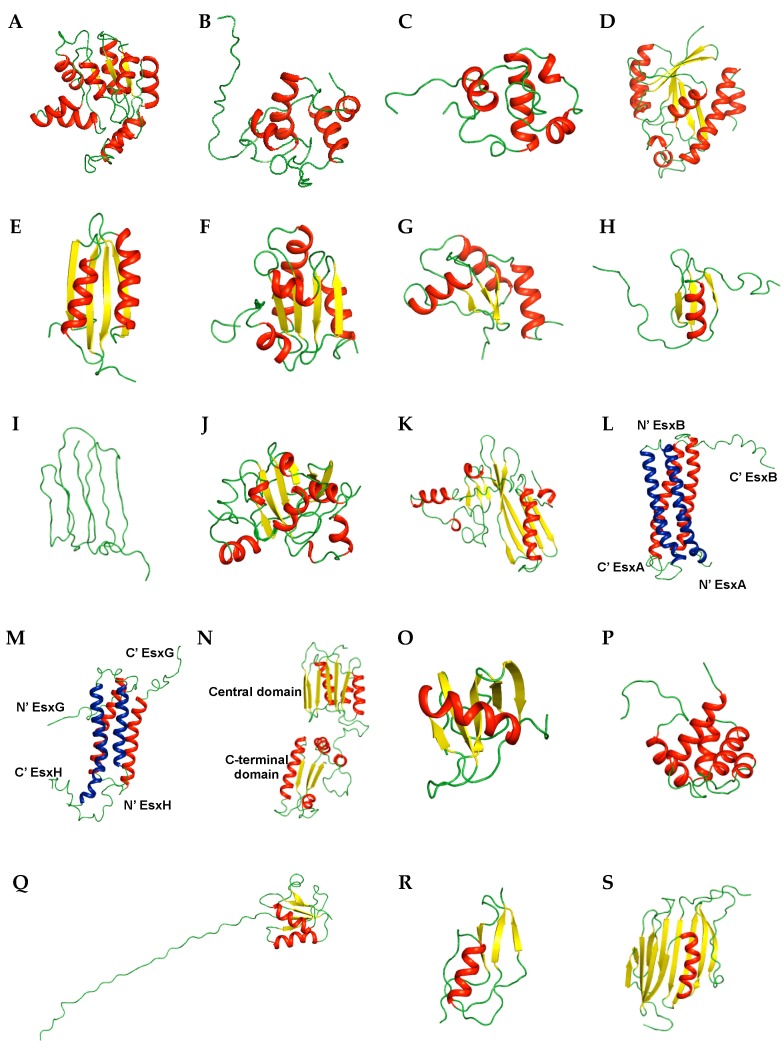
Ribbon representation of NMR structures of *M*. *tuberculosis* proteins. Enzymes and related proteins (**A**) Rv0733 (PDB ID 1P4S); (**B**) Rv1009 (PDB ID 1XSF); (**C**) Rv1884c (PDB ID 2N5Z); (**D**) Rv1014c (PDB ID 2JRC); (**E**) MT1859 (PDB ID 2LQJ); (**F**) Rv3914 (PDB ID 2L59); (**G**) Rv3198.1 (PDB ID 2LQQ). Siderophore-related proteins (**H**) Rv2377c (PDB ID 2KHR); (**I**) Rv0451c (PDB ID 2LW3). Secreted proteins (**J**) Rv2875 (PDB ID 1NYO); (**K**) Rv1980c (PDB ID 2HHI); (**L**) Rv3875/Mb3904 (PDB ID 1WA8); (**M**) Rv0287/Rv0288 (PDB ID 2KG7). Membrane proteins (**N**) Rv0899 (PDB ID 2L26). Uncharacterized proteins (**O**) Rv2302 (PDB ID 2A7Y); (**P**) Rv0543c (PDB ID 2KVC). Other proteins (**Q**) Rv0431 (PDB ID 2M5Y); (**R**) Rv3682 (PDB ID 2MGV); (**S**) Rv2171 (PDB ID 2NC8). The same colors as used in Figure 1 are employed. Two helix-turn-helix hairpins of (**L**) and (**M**), originated from different proteins were colored in blue (EsxA (**L**) and EsxH (**M**) and red (EsxB (**L**) and EsxG (**M**)), respectively.

Overall structures of target proteins provide information for understanding the mechanisms of action and binding sites, as well as others. With various NMR techniques, biochemical studies can be conducted. Thus, the solution structures of several target proteins from *M*. *tuberculosis* have been studied using NMR spectroscopy. We categorized the proteins structures according to their functions in Table 1. Representative structures are shown in Figure 1 and Figure 2, and the details are introduced below.

### 2.1. Transport-Related Proteins

The first structure of an *M*. *tuberculosis* protein determined by solution NMR was Rv2244, the acyl carrier protein AcpM, in 2002 [9]. Acyl carrier proteins (ACPs) transport intermediates between type II fatty acid synthases [10]. As *M*. *tuberculosis* produces extremely long mycolic acids, AcpM has a unique fold and is composed of a folded amino-terminus and highly flexible carboxyl terminus [11]. The topology of AcpM is “square” [12] comprising four α-helices that form a right-turn helical bundle (Figure 1A). The carboxyl-terminus of AcpM known as a “molten domain” showed increased mobility as demonstrated by decreased {^1^H}–^15^N HetNOE values and solvent exchange/accessibility data.

Rv3250c, also known as rubredoxinB, is a small nonheme iron-binding protein involved in the electron-transfer process [13]. Because of their roles in electron-transfer processes, rubredoxin proteins are considered potential drug targets for *M*. *tuberculosis*. The overall structure of Rv3250c consists of one three-stranded antiparallel β-sheet ordered in β3-β1-β2 and a 3_10_-helix connected to the N-terminus of β3 (Figure 1B), as well as a zinc that is tetrahedrally coordinated to the S atom of four cysteine residues (Cys9, Cys12, Cys42 and Cys45). This structure containing Cys4-type metal coordination and a three-stranded antiparallel β-sheet is common in rubredoxin proteins associated with various metals [14,15,16,17].

*sulp* is related to the transport of the SO_4_^2−^, and most bacterial SulP proteins and eukaryotic SulP/SLC26 proteins include a C-terminal cytoplasmic sulfate transporter anti-σ factor antagonist (STAS) domain [18]. As the SulP/SLC26 anion transporter, the STAS domain of Rv1739c (residues 437–560) adopts an anti-σ factor antagonist fold containing five α-helices and a four-stranded parallel β-sheet (Figure 1C) [19].

### 2.2. Transcription-Related Proteins

*M*. *tuberculosis* encodes approximately 10 arsenic repressor (ArsR) family proteins as one of the metallo-regulatory proteins that modulate the transcription of metal ion transporters [20,21]. ArsR proteins play roles in the expression of genes encoding proteins related to metal ion detoxification, sequestration, efflux, and possibly other processes [22]. In *M*. *tuberculosis*, two structures of ArsR family regulators, Rv1994c, CmtR [23] and MT3852, NmtR from *M*. *tuberculosis* strain *CDC 1551*/*Oshkosh* [24], were determined by NMR. As metal-responsive transcriptional regulators, CmtR and NmtR are DNA-binding repressors that sense cadmium and nickel, respectively. Their overall structures form a symmetric dimer and exhibit the typical core winged-helix fold of the ArsR family. The topology of the monomer includes five α-helices and two β-sheets arranged in a α1-α2-α3-α4-β1-β2-α5-fold (Figure 1D,E). As the DNA recognition helix, helix α4 is also denoted as αR, and the β-wing tip is structurally less well-defined than the core of the molecule. NmtR has distinct long flexible N- and C-terminal tails compared to CmtR.

Rv0639, also known as N-utilization substances G (MtNusG), is a small molecule regulator that modulates the movement of RNA polymerase (RNAP) along DNA [25,26]. NusG is essential in Gram-negative bacteria, and an inhibitor of the NusG-RNAP interaction may be a drug target [27]. The solution NMR structure of MtNusG-CTD (residues 178–238) is composed of five β-strands folded into an antiparallel barrel-type β-sheet in the order of β5-β1-β2-β3-β4 (Figure 1F). Additionally, to investigate transient domain interactions, titration of unlabeled MtNusG-CTD into ^15^N-labeled MtNusG-NTD was conducted, but there was no significant change in the spectrum. For further analysis, ^15^N relaxation measurements were performed. Because the R_1_ and R_2_ rates are sensitive to the tumbling of proteins and are related to the rotational correlation time, the R_2_/R_1_ ratio indicates molecular reorientation in solution [26]. The calculated R_2_/R_1_ ratio of NusG showed a different distribution of residues from MtNusG-CTD and MtNusG-NTD, indicating a different reorientation on the timescale of molecular rotation. Thus, there are no tight domain interactions for MtNusG.

Rv2050 is RNAP-binding protein A (RbpA) and affects transcription by binding to the β subunit of RNAP. It is also related to the response of *M*. *tuberculosis* to the anti-TB drug rifampicin [28]. RNAP contains a core of five subunits (α_2_ββ′ω) and a sixth σ-subunit (or σ factor) which is involved in promoter recognition and transcription initiation [28,29]. Hu et al. found that stabilizing the formation of the RNAP holoenzyme containing σ^A^ by RbpA activates transcription [30]. These roles of RbpA such as gene expression regulation, normal growth of *M*. *tuberculosis,* and rifampicin tolerance make RbpA as a drug target. The structure of RbpA (residues 1–79) comprises four β-strands forming two antiparallel β-sheets connected by turns and loops (Figure 1G). The two β-sheets appear as a β-sandwich-like structure and are stabilized by aromatic and non-polar residues (Tyr32, Val42, Phe44 and Trp54).

### 2.3. Nucleotide-Binding Proteins

The PhoP-PhoR (PhoPR) two-component system of *M*. *tuberculosis* is involved in microbial adaptation [31]. PhoP is known to regulate numerous genes related to cellular functions and is involved in *M*. *tuberculosis* virulence [32]. Therefore, understanding its structure and function is important. The J113_05350, PhoP protein from *M*. *tuberculosis CAS*/*NITR204*, has two folded domains, an N-terminal receiver domain phosphorylated by PhoR (PhoPN; residues 1–138) and C-terminal effector domain that binds DNA (PhoPC; residues 150–247) [33]. The structure of the PhoPC DNA-binding domain was evaluated to determine the mechanism of DNA binding and the structural role of the interdomain linker [34]. The overall structure of PhoPC (residues 142–247) consists of two β-sheets packed against a three-helix bundle in the order of β6-β7-β8-β9-α6-β10-α7-α8-β11-β12 (Figure 1H). The elevated mobility of the loop connecting α7 and α8 (α7/α8 loop, residues 206–211), N-terminus, and K230 and V239 was observed from the {^1^H}–^15^N-HetNOE and RCI-S^2^ data.

Rv3597c, also known as Lsr2, consists of an N-terminal dimerization domain and C-terminal DNA-binding domain [35]. As a nucleoid-associate protein, Rv3597c binds to AT-rich sequences and various genes related to virulence, antigenicity, and cellular processes such as DNA replication, transcription, and protein synthesis [36]. When *lsr2* is up-regulated under hostile conditions within the host, inhibition of the expression of the above genes allows *M*. *tuberculosis* to remain latent [36]. Lsr2 is thought to be a unique drug target for controlling latent tuberculosis infection. NMR was used to solve the structure of the C-terminal DNA-binding domain of Lsr2 (Lsr2C, residues 66–112), which is composed of two α-helices (α1, residues 78–89; α2, residues 102–112) connected by a long loop and packed through hydrophobic interactions (Figure 1I).

### 2.4. Ser/Thr Protein Kinase-Related Proteins

*M. tuberculosis* expresses a eukaryotic like kinase-signaling network consisting of 11 predicted Ser/Thr protein kinases (STPKs) (named as PknA-PknL, except for PknC) [37], at least one Ser/Thr phosphatase (PstP) and two PTPs (PtpA and PtpB) [38], and five pThr-binding Forkhead-associated (FHA) domain-containing proteins [39]. These proteins are involved in diverse stages of growth, development, and pathogenesis in *M*. *tuberculosis* [40]. Thus, STPKs and related proteins are attractive targets for inhibitor development. There have been several NMR studies of STPK, phosphatase, and FHA domain functions to reveal structural information for the proteins and the signaling pathways of *M*. *tuberculosis*.

Because of its importance in mycobacterial growth, PknB is considered a drug target [41]. Rv0014c, PknB is a transmembrane STPK and possesses extracellular penicillin and Ser or Thr kinase-associated (PASTA) domain, which is a signaling molecule [42]. To investigate the regulation of PASTA domain-mediated PknB activity, structural studies were conducted using NMR [43]. Because of the large size of the PASTA domain, structure determination was conducted using the proteins dissected into three overlapping bidomain constructs: PknB_PASTA12 (residues 354–491; PDB ID 2KUD), PknB_PASTA23 (residues 423–557; PDB ID 2KUE), and PknB_PASTA34 (491–626; PDB ID 2KUF), corresponding to domains 1–2, 2–3 and 3–4, respectively. The overall secondary structure topology was β′-α1-β1-β2-β′′-β3. Based on these structures and small-angle light scattering experiments, the overall structure of the PASTA domain was modeled (PDB ID 2KUI) (Figure 1J).

As substrates of PknB, Rv1827 and Rv0020c, which have FHA domains, were studied. Rv1827 is specifically phosphorylated at Thr22 within a conserved N-terminal T_21_T_22_SVF motif that is the preferred PknB target sequence [44]. To investigate the regulation mode of the FHA domain at the molecular level, solution structures of Rv1827 and Rv1827-pThr22 were determined using multidimensional heteronuclear NMR [45]. Rv1827 contains a classical FHA domain fold comprising an 11-stranded β-sandwich topology (Figure 1K). The conserved residues, Arg81 and Ser95, contact pThr22. The linker region (residues 34–54) and extreme N- and C- termini of Rv1827 were not well-defined. ^15^N relaxation data represented by {^1^H}–^15^N HetNOE, T_1_, and T_2_ values also showed increased internal mobility of these regions. The dynamic behavior of the region containing pThr22 exhibited similar characteristics to the core FHA domain. Rv0020c contains 527 amino acid sequences. For structural and interaction studies, the N-terminal domain (PDB ID 2LC0, residues 1–132) and FHA domain (PDB ID 2LC1, residues 430–527) structures were determined [46]. The N-terminal domain contains a three-stranded β-sheet faced on one side by two amphipathic α-helices (α1, α3) with a topology of α1-β3-α3-β1-β2. This αβ sandwich is capped by the short helix α2 (Figure 1L). The FHA domain of Rv0020c also adopted the global FHA domain fold composed of an 11-stranded β-sandwich topology (Figure 1L).

The structure of Rv2175c as a substrate of PknL was also determined [47]. Rv2175c has two domains: an N-terminal winged helix-turn-helix motif (residues 18–77), a DNA-binding domain, and C-terminal effector domain (residues 78–146), and contains six α-helices and two β-strands (Figure 1M). There is some flexibility between the two domains. The solution structure of Rv2175c revealed that the N-terminal part (Met1–Ile16) and large loop (Thr110–Asn122) were disordered. The chemical shift perturbations of Rv2175c according to the phosphorylation of Thr9 showed no significant structural change in the monomer, but electrophoretic mobility shift assay experiments showed that the phosphorylation of Thr9 negatively affected its DNA binding.

Rv2234 is a low-molecular weight protein-tyrosine phosphatase (LMW-PTP) and is also known as MptpA. Because Rv2234 is essential for the survival of *M*. *tuberculosis* upon infection of host macrophages, it is considered a drug target [48]. The structure of Rv2234 exhibits a Rossman fold which is common in LMW-PTPs containing a four-stranded parallel β-sheet connected via five α-helices in the order of β1-α1-β2-α2-α3-β3-α4-β4-α5 (Figure 1N) [49]. The highly conserved active site of LMW-PTPs involves the residues Cys11-Thr-Gly-Asn-Ile-Cys-Arg-Ser18 positioned in a loop known as the phosphate-binding loop (P-loop), and the catalytic active residues is Cys11. The β2-α2 loop containing residue Trp48 (W-loop, Gly44–Asp55) and β4-α5 loop containing critical residue Asp126 (D-loop, Arg111–Asp131) are positioned close to the P-loop. Additionally, Trp48 and His49 are involved in substrate specificity and Asp126 is crucial for the catalytic mechanism.

### 2.5. Enzymes and Related Proteins

Rv0733 is known as an adenylate kinase (AK), which is involved in energy metabolism and nucleic acid synthesis [50]. As an essential enzyme in metabolism and because of its unique catalytic properties [51], AK is considered a novel target for anti-TB drugs. The protein is composed of nine α-helices and five β-strands and contains an ATP-binding motif that binds the phosphates of ATP (Figure 2A) [52]. This motif is termed as the P-loop and is represented as GXXGXGK which ranges from residues 7 to 13 in Rv0733.

Resuscitation-promoting factor (RPF) proteins is related to normal proliferation of dormant bacteria and is involved in reactivation of stationary-phase cultures of (G + C)-rich Gram-positive bacteria, including *M*. *tuberculosis* [53,54]. Therefore, understanding the functional activity of RPF proteins will provide an insight for the development of anti-TB drugs. *M*. *tuberculosis* encodes five RPF proteins (A–E) [55] and two RPF proteins among them, Rv1009 (also known as RpfB) [56] and Rv1884c (also known as RpfC) [57], have been studied using NMR. The solution structure of the C-terminal 108 residue core domain of RpfB (RpfBc) is composed of five α-helices (Figure 2B). RpfBc shares high structural homology with c-type lysozymes (PDB ID 3LZT, *Z*-score of 6.4, r.m.s. deviation of 2.9 Å over 79 residues) [58]. The solution structure of the RpfC catalytic domain consists of four α-helices with an N-terminal flexible tail (Figure 2C). The catalytic residue Glu16 is positioned on the α1 helix. The unstructured C-terminus is anchored to the N-terminus of the domain through a disulfide bond (Cys15–Cys76).

Rv1014c, also known as MtPth, is peptidyl-tRNA hydrolase (Pth) [59]. The unique properties and importance of Pth in bacteria and synergistic effect of Pth inhibitors with macrolide antibiotics suggest Pth is an attractive antibiotic target [60]. Rv1014c consists of central seven β-sheet encompassed by six α-helices and additional two 3_10_ helices (3_10_–1 and 3_1_–2) (Figure 2D). The catalytic base His22 of MtPth is positioned in a short segment between the 3_1_–1 helix and α1 and is exposed on the surface of the structure. The backbone dynamics measured by ^15^N relaxation experiments revealed overall rigidity of this enzyme with τ_m_ of 9.67 ± 0.02 ns. The segments between the 3_10_–1 helix and α1 and C-terminal helical hairpin of the protein undergo ms to μs timescale motions and may be related to the interaction with the substrate peptidyl-tRNA.

MT1859 from *M*. *tuberculosis strain CDC 1551*/*Oshkosh*, also known as MgtC, is a virulence factor related to survival inside macrophages in *M*. *tuberculosis* as other intracellular bacterial pathogens [61]. The structure of the C-terminal domain adopts the fold of an aspartokinase, chorismate mutase, and TyrA (ACT) domain which is a small molecule-binding domain comprised of a βαββαβ fold (Figure 2E) [62].

The thioredoxin system is essential for redox homeostasis and maintains cellular proteins in a reduced state [63]. The thioredoxin system in *M*. *tuberculosis* is composed of three thioredoxins (TrxA, TrxB, and TrxC) and one thioredoxin reductase (TrxR), in which Trx becomes oxidized, and is reduced by TrxR [64]. Because of the low similarity (35%) of *M*. *tuberculosis* thioredoxin with human thioredoxin and its importance in oxidative stress, inhibitors selectively targeting the thioredoxin system in *M*. *tuberculosis* may be developed as anti-mycobacterial drugs [65]. In this context, Rv3914, TrxC was studied using NMR [65]. The structures of TrxC were determined in two states: oxidized (PDB ID 2L59) and reduced (PDB ID 2L4Q). TrxC in both states contains a common fold of four α-helices surrounding a five stranded β-sheet core composed of three parallel strands and two antiparallel strands (Figure 2F).

Rv3198.1, known as mycoredoxin-1 (Mrx1), is mycothiol-dependent reductase. As a novel member of the Trx-family, Mrx1 uses the MSH electron transfer pathway to reduce disulfides in *M*. *tuberculosis* and is related to several external stresses including oxidative stress, alkylating agents, and antibiotics [66,67]. The structure of Mrx1 was determined in two states, oxidized (PDB ID 2LQQ) and reduced (PDB ID 2LQO) [67], and contains a thiroredoxin-fold composed of a four-stranded antiparallel β-sheet encompassed by three α-helices (Figure 2G), which is found in several oxidoreductases [68]. TrxC and Mrx1 have a redox active CXXC motif [68] at the N-terminus of α2 (Cys37-Gly-Pro-Cys40) and α1 (Cys14-Gly-Tyr-Cys17), respectively, and a conserved proline opposite to the active site is present in the *cis* conformation.

### 2.6. Siderophore-Related Proteins

Because iron is an essential nutrient in most bacterial pathogens, disruption of the biochemical pathway for the sequestration of iron may be a target for novel antibiotics [69,70]. *M*. tuberculosis uses two siderophores, mycobactins and carboxymycobactins, to scavenge iron from the human host [71,72]. Two siderophore-related proteins have been studied by NMR in *M*. *tuberculosis*.

Rv2377c, MbtH, is a small, 71-residue protein, and one of the proteins composing non-ribosomal protein synthetase clusters involved in siderophore and antibiotic peptide synthesis [73]. The structure of Rv2377c is composed of a three-stranded anti-parallel β-sheet ordered in β3-β1-β2 and one C-terminal α-helix (Figure 2H) [74]. Dynamic experiments revealed the μs to ms time scale motion of the interface between β1–β2 and disordered C-terminus region. This dynamic property of the regions containing conserved residues of MbtH-like family may be associated with diverse biological functions and various specific substrates [74].

Rv0451c, known as MmpS4, interacts with MmpL4 which transports small molecules. MmpS4/MmpL4 acts as a siderophore export system in *M*. *tuberculosis* [75]. The structure of the C-terminal soluble domain Rv0451c (residues 52–140) contains seven consecutive β-strands arranged in two layers, with β4-β1-β6-β7 in one layer and β3-β2-β5 in the other layer (Figure 2I). Interestingly, Rv0451c shows no similarity to proteins with known function such as periplasmic adapter proteins from drug efflux systems of Gram-negative bacteria [76], except for an uncharacterized protein from *Parabacteroides distasonis* (PDB ID 2LGE). This indicates that MmpS4 represents a novel class of accessory proteins in complex transporter systems.

### 2.7. Secreted Proteins

Because secreted proteins from *M*. *tuberculosis* are targeted by the host immune system, the proteins have been investigated for vaccine development and immunodiagnostics [77]. Several secreted proteins from *M*. *tuberculosis* have been studied by NMR spectroscopy.

There are two reported solution structures of immunogenic proteins secreted from *M*. *tuberculosis*. Rv2875, known as immunogenic protein MPT70, folds into a seven-stranded β-barrel flanked by eight α-helices (Figure 2J) [78]. Rv2785 shares structural homology with FAS1 domains 3 (Fas3) and 4 (Fas4) of fasciclin Ι (PDB ID 1O70) [79]. Based on the sequence and structural homology with FAS1 domains from extracellular protein (fasciclin Ι and *β*ig-h3) that are involved in the interactions between cell surface and extracellular matrix proteins supports the role of Rv2875 in binding to host cell surface proteins [78].

Rv1980c, known as MPT64, has a pseudo-two-domain and adopts a novel fold containing a β-grasp-like motif in the N-terminal region which includes long α-helix (residues 42–60) contacts at a shallow angle against a plane formed from a seven-strand β-sheet (Figure 2K) [80]. Rv1980c antigen is currently utilized to detect active TB infection [81,82].

*M*. *tuberculosis* utilizes five type VII secretion systems to export several proteins such as Esx family members [83]. Esx proteins are related to TB pathogenesis and survival in host cells, and are associated with metal ion acquisition [84,85,86,87]. Several Esx pairs such as including EsxA/EsxB, EsxG/EsxH (Rv0287/Rv0288), EsxR/EsxS (Rv3019c/Rv3020c), and EsxO/EsxP (Rv2346c/Rv2347c) form tight complexes in their functional forms [88,89,90]. Among them, the structure of EsxA/EsxB (Rv3875/Mb3904 from *M*. *bovis*) (Figure 2L) [91] and EsxG/EsxH (Rv0287/Rv0288) (Figure 2M) [92] were studied by NMR. The overall structure of both complexes forms a 1:1 heterodimer and is made up of two similar helix-turn-helix hairpin structures originating from individual proteins. To form a four-helix bundle, proteins utilize an extensive hydrophobic contact surface lying antiparallel to each other. Commonly, the long flexible arms at N- and C-termini of both proteins are disordered. However, structural distinctions between two complexes exist. EsxG and EsxH have shorter helices at the N-terminus than EsxA and EsxB, and EsxB shows a helical conformation at the C-terminus. Additionally, EsxG/EsxH has a specific Zn^2+^ binding site detected by NMR titration experiments on a noticeable cleft as a functional site, unlike EsxA/EsxB. These features contribute to the distinct functional roles of two Esx family complexes; EsxA/EsxB is involved in pathogen-host cell signaling and EsxG/EsxH has been implicated in metal ion scavenging [87,90,92].

### 2.8. Membrane Proteins

Rv0899, also known as OmpATb, has been reported to be a pore-forming protein, and under low pH conditions is involved in the permeability of several small, water-soluble molecules [93,94] and self-defense mechanism through ammonia secretion [95]. Additionally, expression of OmpA only in pathogenic species support its role in the virulence of these mycobacterial strains [94]. To investigate the roles of Rv0899, several structural studies using NMR have been conducted [96,97,98,99] with various constructs. Rv0899 is divided into three domains: N-terminal domain (residues 1–72) including a membrane-anchoring sequence, central domain (residues 73–200), and C-terminal domain (residues 201–326). The structure of the central domain shares homology with the BON (bacterial OsmY and nodulation) superfamily (pfam04972) [100], containing a six-stranded parallel/antiparallel β-sheet facing three parallel/antiparallel α-helices (Figure 2N). The C-terminal domain consists of four α-helices and four β-strands with αβαβαβαβ topology (Figure 2N) that is the typical α/β-structure of peptidoglycan-binding domains in the OmpA-like superfamily (pfam00691) [101].

### 2.9. Uncharacterized Proteins

Rv2302 is comprised of 80 amino acids and has an unknown function, but is highly conserved in *M*. *tuberculosis* [102]. The solution structure of Rv2302 folds into a five-strand antiparallel β-sheet with a β-sheet twist flanked by a C-terminal α-helix (Figure 2O). The absence of 2D ^1^H–^15^N HSQC crosspeaks for residues between the β1 and β2 (loop L1) revealed an ms to μs timescale motion of the loop Ll region. {^1^H}–^15^N-HetNOE results revealed that the overall structure was rigid except for the N- and C-termini. In the surface representation, a positively charged pocket composed of Arg18, Arg70, and Arg74 can be observed. As Arg18 is positioned in loop L1 which shows intermediation motion, Arg18 is considered to play a role in the biochemical function of Rv2302.

Rv0543c is the protein categorized in the Domain of Unknown Function (DUF) and belongs to the DUF3349 (PF11829) superfamily [103]. The structure which was determined by NMR is composed of a bundle of five α-helices containing highly conserved hydrophobic residues inside (Figure 2P). The backbone dynamics revealed by ^15^N relaxation experiments showed the restricted flexibility of overall residues except the unstructured C-terminus.

### 2.10. Other Proteins

Rv0431 is involved in regulating membrane vesicles of *M*. *tuberculosis* and is known as the vesiculogenesis and immune response regulator (VirR) [104]. Following the deletion of highly hydrophobic residues, the truncated protein (residues 42–164) structure was intrinsically disordered in the first 35 amino acids in the N-terminus, and the residual ordered region (residues 78–164) adopted a unique fold containing a five-stranded β-sheet packed by two α-helices on one side and one α-helix on the other (Figure 2Q). Although the mechanism of how VirR regulates vesiculogenesis remains unclear, an intrinsically unstructured region of VirR may be related to the interaction with other proteins.

Rv3682, annotated as PonA2, is one of the two class A penicillin binding proteins (PBPs) of *M*. *tuberculosis*. PBPs are enzymes involved in the synthesis, maturation, and recycling of peptidoglycan, which is an essential building block of the bacterial cell wall [105]. Additionally, a C-terminal PASTA domain of PonA2 is related to the adaptation of *M*. *tuberculosis* to dormancy [106]. For these properties, Rv3682 is considered an attractive target for inhibitors. The solution structure of the C-terminal PASTA domain of PonA2 (PonA2-PASTA, residues 700–764) exhibits typical PASTA domain topology comprising the N-terminal α-helix followed by a three stranded β-sheet arranged in order of β1-β3-β2 (Figure 2R). Interestingly, PonA-PASTA shows a higher structural similarity with PASTA domains of STPKs than with the PBP2x family [106].

Rv2171, LppM (residues 26–185) has 11 β-strands, a pseudo β-barrel closed by an α-helix, and a degenerated β-strand (Figure 2S) [107]. Although LppM is known as a lipoprotein, it does not share homology with other known lipoproteins from *M*. *tuberculosis*, defining a new protein fold. This protein is related to the phagocytosis and efficient blocking of phagosomal acidification in macrophages, resulting in *M*. *tuberculosis* persistence [108], and can bind *M*. *tuberculosis* phosphatidylmyoinositol mannoside, which is known to affect the host immune response [107].

## 3. NMR-Based Molecular Interaction

Understanding the molecular mechanism of action from binding information may provide insight for the development of novel antibiotics. Several experimental NMR approaches have been developed to observe interactions between proteins and ligands in solution [109]. Using NMR-based methods, changes in the spectra of the protein or the ligand can be monitored to observe molecular interactions.

### 3.1. Protein-Observed NMR for Interaction Mode

NMR spectroscopy can be applied for monitoring interactions between target proteins and various molecules. Among several methods, chemical shift perturbation (CSP) analysis is the most widely used technique because of the high sensitivity of 2D ^1^H–^15^N HSQC experiments [110]. Additionally, this method typically requires relatively low concentrations and short recording times. Every residue except for proline contains an amide atom, and 2D ^1^H–^15^N HSQC data reflect the chemical environment of all parts of the protein. Addition of unlabeled molecules (proteins, metal, nucleic acid) to ^15^N-labeled proteins alter the chemical environment, resulting in a chemical shift change in the spectrum. Mapping the altered residues on a known structure can provide information such as the binding interface. In studies of proteins from *M*. *tuberculosis*, interaction modes with various molecules were detected based on protein-observed NMR methods. Below, we describe some examples.

#### 3.1.1. Rv2050, RbpA

To understand the relationship between full-length RbpA and σ subunits, 2D ^1^H–^15^N TROSY and 3D ^1^H–^13^C–^15^N TROSY-HNCO spectra of isotope-labeled RbpA and isotope-labeled RbpA bound to unlabeled His_6_-σ^B^_1–228_ were recorded [28]. The backbone amide groups showing evident signal changes by His_6_-σ^B^_1–228_ binding included Ala13, Ser15, Tyr16, and 10 unassigned residues. The unassigned RbpA backbone amide groups belonged to the C-terminal region (residues 77–111). This binding experiment indicated that the central domain of RbpA is not directly affected by formation of the complex with His_6_-σ^B^_1–228_, but the N- and C-termini are related to binding. RbpA forms a tight complex with His_6_-σ^B^_1–228_ via its N- and C-terminal regions.

#### 3.1.2. Rv1739c

**Figure 3:**
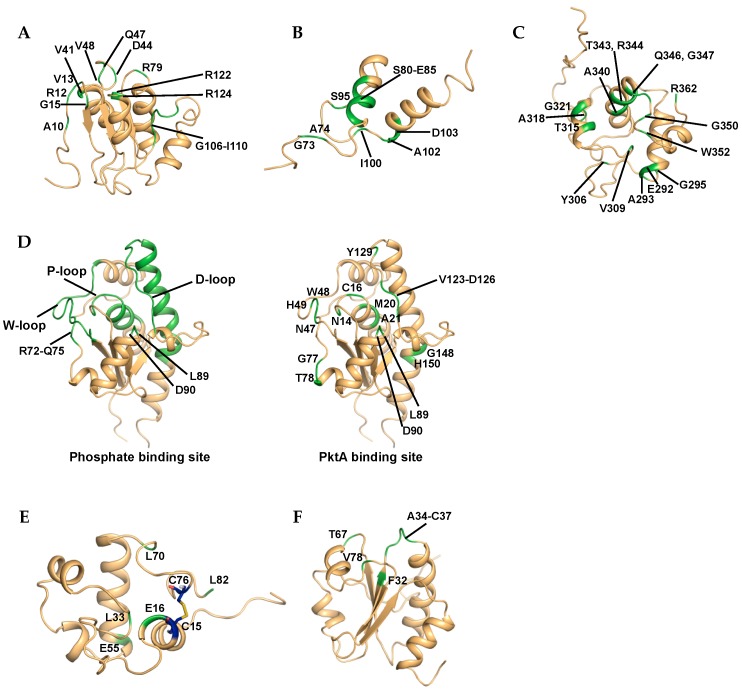
The mapping of the binding and conformational change sites on the ribbon representation of *M*. *tuberculosis* proteins. (**A**) GDP binding site of Rv1739c (PDB ID 2KLN); (**B**) DNA binding site of Rv3597c (PDB ID 2KNG); (**C**) tri-NAG binding site of Rv1009 (PDB ID 1XSF); (**D**) Phosphate binding site (Left) and PktA binding site (Right) of Rv2234 (PDB ID 2LUO); (**E**) Conformational change site by pH of Rv1884c (PDB ID 2N5Z); (**F**) TrxR binding site of Rv3914 (PDB ID 2L59). The residues showing significant chemical shift changes are labeled and colored in green. The disulfide bond is represented in blue color.

The interaction of the STAS domain of Rv1739c with GTP or GDP was investigated in 2D ^1^H–^15^N HSQC experiments [19]. GDP binding showed chemical shift changes of 16 residues, including Ala10, Arg12, Val13, Gly15, Val41, Asp44, Gln47, Val48, Arg79, Gly106, Glu107, Asp108, His109, Ile110, Arg122, and Arg124 (Figure 3A). GTP also affected the chemical shifts of residues Ala10, Arg12, Val13, Gly15, Val48, Val61, Arg79, His109, and Arg124. CSP analysis of titration experiments revealed nucleotide-induced conformational perturbations at solvent exposed loops and close residues.

#### 3.1.3. Rv3597c, Lsr2

To monitor the DNA-binding site of Lsr2C, a 27-mer DNA containing nine consecutive A-T base pairs was used in titration experiments utilizing 2D ^1^H–^15^N HSQC spectra [36]. Residues showing significant CSP values were Gly73, Ala74, Ser80–Glu85, Ser95, Ile100, Ala102, and Asp103, and surface mapping showed that the residues were located predominantly on the α1 helix and nearby linker loop (Figure 3B). Based on the results, the Lsr2C·DNA complex structure was predicted using HADDOCK 2.0 [111], and DNA binding of Lsr2c occurred by grabbing either edge of the minor groove of DNA like a clamp.

#### 3.1.4. Rv1009, RpfB

To analyze the binding of RpfBc with oligosaccharide, NAG and its trimer, *N*,*N*′,*N*′′-triacetyl-chitotriose (tri-NAG) were evaluated in NMR titration experiments [56]. The binding NAG monomer exhibited no chemical shift changes, but the addition of tri-NAG resulted in chemical shift changes at residues in the putative binding groove, including the loop connecting α4 and α5 (Figure 3C). Gln347 was the most affected residue and an equivalent conserved glutamine in the lysozyme (Gln104) was a key residue for binding oligosaccharide.

#### 3.1.5. Rv2234, MptpA

The binding of MptpA with phosphate ion (Pi) as a competitive inhibitor was investigated by comparing 2D ^1^H–^15^N TROSY data with and without NaH_2_PO_3_ [49]. Based on the results, six residues (Asn14, Cys16, Trp48, Asp90, Tyr128, and Tyr129) located in the P-loop disappeared because of line broadening. CSP analysis revealed five regions affected by phosphate binding to the active site: Asn14–Met24 (P-loop), Ala43–Glu56 (W-loop), Arg72–Gln75, Lys89 and Asp90, and Asp123–His150 (D-loop and hinge region in α5-helix) (Figure 3D). Phosphate titration experiments showed that the functionally important P-, W-, and D-loop as well as other spatially related regions are involved in the specific binding of phosphate to MptpA.

As MptpA is phosphorylated by the kinase PtkA [112], to understand the binding interface of the MptpA·PtkA complex, a titration experiment was conducted. CSPs were observed during NMR titration analysis of ^15^N-labeled MptpA with unlabeled PtkA. The backbone amides of MptpA showing the largest CSP values upon binding of PtkA were Asn14, Cys16, Met20, Ala21, Asn47, Trp48, His49, Gly77, Thr78, Leu89, Asp90, Val124, Glu125, Asp126, Tyr129, Gly148, and His150. Amide resonances of Cys16 and Trp48 were not detected. Mapping of CSPs onto the structure indicated that MptpA·PtkA complex interface contains P-, W-, and D-loop motifs with additional regions, including the residues Met20, Gly77, and Asp90 as well as Ala145 (Figure 3D).

#### 3.1.6. Rv1884c, RpfC

RpfC function via a redox-sensing switch mediated by cysteine residues was assessed using NMR [57]. 2D ^1^H–^15^N HSQC spectra of Rpfc were acquired at pH 7, 5 and 3 in the presence of TCEP to maintain reducing conditions. This experimental condition mimicked the hydrolysis of the disulfide bridge between Cys15 and Cys76. Significant chemical shift changes were observed for residues surrounding the disulfide bond and the residues Leu33, Glu55 and Leu7, which are far from the cysteine residues (Figure 3E). These data indicate that the oxidation state of the cysteine residues modulates the shape of the hydrophobic catalytic cleft via conformational changes.

#### 3.1.7. Rv3914, TrxC

To understand the interaction between TrxC and TrxR, 2D ^1^H–^15^N HSQC spectra of ^15^N-labeled TrxC was monitored following the addition of TrxR which was reduced by NADP(H) or DTT [65]. The crosspeaks corresponding to the TrxR binding interface residues of TrxC either disappeared or showed large chemical shift changes. The residues were Phe32, Ala34, Thr35, Trp36, Cys37, Thr67, and Val78, and these residues form a hydrophobic surface (Figure 3F). Based on the TrxC-TrxR binding interface information obtained from NMR experiments, inhibitor screening was conducted by docking simulation. Among the 10,000 drug-like chemicals, a high scoring compound was selected. Binding was confirmed using 2D ^1^H–^15^N HSQC. Compound CSDDD_6702 showed chemical shift perturbations of several residues including the interface residue Trp31 (HE1). These results provide insight for the design of inhibitors for treating TB.

#### 3.1.8. Rv3682, PonA2

As a penicillin binding protein, PonA2-PASTA was titrated with β-lactam antibiotics (cefuroxime and cefotaxime) [106]. The cross peaks of 2D ^1^H–^15^N HSQC exhibited no significant chemical shift perturbations, so it was concluded that PonA2-PASTA is not involved in β-lactam antibiotic binding. To validate whether PonA2-PASTA is involved in a muropeptide-sensing mechanism, two muropeptides typically used to mimic the peptidoglycan peptide stem, L-Ala-γ-D-Glu-mDAP and MurNAc-L-Ala-γ-D-Glu-mDAP were used in titration experiments. No significant interactions between PonA2-PASTA and muropeptides were observed.

### 3.2. Ligand-Observed NMR for Drug Discovery

Ligand-based NMR techniques can be applied to detect protein-ligand interactions. This method is efficient because: (i) target protein labeling such as by ^13^C or ^15^N is not necessary and (ii) small amounts of proteins are needed compared to protein-based NMR. Several methods can be used to monitor changes in the small molecule spectra: saturation transfer difference spectroscopy (STD) [113] and Water-LOGSY [114], which are NOE effect-based experiments, and DOSY [115], which is a diffusion based experiment.

Among them, STD is a popular ligand-based NMR technique for observing molecular interactions. Selective saturation of a resonance belonging to the protein is applied to the sample. If the ligand binds, saturation propagates from the selected protein protons to other protons of the protein via spin diffusion and then saturation is transferred to binding compounds by cross-relaxation at the ligand-protein interface [113]. The STD method has been used to characterize the protein-ligand complex. This method can be applied for screening of the efficient ligands as therapeutic targets.

Using several biophysical techniques such as thermal shift screening, isothermal titration calorimetry, and X-ray crystallography, target enzymes from *M*. *tuberculosis* have been studied to identify inhibitors using ligand-based NMR methods.

#### 3.2.1. Rv3809c, UDP-Galactopyranose Mutase (UGM)

UDP-galactopyranose mutase (UGM) catalyzes the interconversion of UDP-Galp and UDP-Galf [116]. Galactofuranose (Galf) is an essential component of galactan chains in the cell walls of mycobacteria [117,118]. Therefore, inhibiting Rv3809c, UGM from *M*. *tuberculosis* (MtUGM) resulting in reduced UDP-Galf is a good strategy for identifying effective anti-TB drugs. Shi et al. revealed a second, druggable binding site of MtUGM using the non-substrate-like UGM inhibitor, MS-208 [119]. The elevated STD signal of the inhibitor indicated binding between MtUGM and MS-208. To investigate the binding site of MS-208, competition STD NMR experiments between MS-208 and UDP-Galp, which binds to the S-site, were conducted. By increasing the concentration of each molecule, STD enhancement decreased, indicating competition between MS-208 and UDP-Galp. However, MS-208 binding could not completely eliminate UPD-Galp and the inverse binding. Thus, MS-208 binds at a second site to directly affect substrate binding. UPD, the active site binding ligand of MtUGM, was used in competitive STD NMR experiments. UDP reduced MS-208 binding by approximately 50%, but MS-208 did not significantly affect UDP binding. Therefore, Shi et al. predicted that UDP-Galp/UDP and MS-208 affect the binding of each other through mutual allosteric effects to different degrees. Additional kinetic assays, computational modeling, and mutant experiments revealed that MS-208 binds to the allosteric binding site (A-site) of MtUGM. The presence of the A-site provides second possible target information for the development of anti-TB pharmaceuticals.

#### 3.2.2. Rv2276, CYP121

The presence of the 20 cytochrome P450 enzymes (CYPs) encoded by the mycobacterium genome suggest the importance of these enzymes. Their relationship with the virulence and survival of mycobacterium also makes CYPs potential targets for the treatment of *M*. *tuberculosis* infections [120,121,122]. Rv2276, CYP121 from *M*. *tuberculosis* is specific to this bacterium and catalyzes the formation of a C–C bond between the two tyrosine residues of the substrate cyclodityrosine (cYY), resulting in the formation of mycocyclosin [123]. Thus, CYP121 is considered the most promising anti-TB drug target among other CYPs. Currently known CYP121 inhibitors with high affinities are azole antifungals that function via a type II azole heme coordination [124,125] Hudson et al. investigated inhibitors targeting CYP121 [126]. Through fluorescence-based thermal shift screening, 66 hits were identified against CYP121 among 665 fragments. 66 chemically diverse hits from a thermal shift assay were further screened by 1D ^1^H-NMR spectroscopy of STD experiments. The STD spectrum of a fragment hit was recorded in the presence and absence of CYP121 or CYP121 plus cYY. Positive signals in the presence of CYP121 indicated the binding of the fragment to the protein, and additional signals for the aromatic region in the presence of CYP121 plus cYY originating from cYY indicated that the fragment-protein interaction is displaced by cYY and that the fragment binds to the active site of CYP121.

## 4. Conclusions

In this review, we analyzed the 50 known solution NMR structures of proteins from *M. tuberculosis* as targets for antibiotics. Structural and functional studies and ligand interactions of these target proteins provide important information for drug discovery. From studies of proteins presented in *M*. *tuberculosis*, information regarding the active site, ligand binding site, and mechanism of action, as well as structures of target proteins, can be obtained.

NMR spectroscopy is powerful tool for screening inhibitors and searching ligands. Compared to X-ray crystallography, size limitations remain problematic, but this method can provide not only structural information, but also biophysical information including protein-ligand binding and dynamics, which cannot be identified using other methods.

Although NMR structure determination is confined to relatively small and medium size molecules, NMR is valuable when studying structurally flexible protein, difficult to be crystallized. NMR spectroscopy can detect the binding interface and properties of target proteins with their diverse binding partner, such as nucleotides, small molecules or metals. Binding affinities can be explained via chemical shift changes between apo- and bound-form. Furthermore, high resolution NMR spectroscopy can provide confident conformations even for uncrystallizable complexes because of low binding affinity.

Identification of unknown ligand-binding sites would contribute to the development of novel active compounds. Conformational dynamics of the binding sites of target proteins determined by NMR will enable identification of lead compounds and the design of potent inhibitors. Particularly, ligand-based NMR techniques, such as STD, Water-LOGSY, and CPMG NMR pulse programs, have attracted attention as methods for identifying efficient lead compounds. Taken together, these NMR studies of target proteins from *M. tuberculosis* will provide a structural basis for developing novel antibiotics.

## Figures and Tables

**Table 1:** Overview of NMR structures from *M*. *tuberculosis* proteins.

Classification	Protein	Brief Description	PDB ID (Deposit Year)
Transport-related proteins	Rv2244	Acyl carrier protein	1KLP (2001)
	Rv3250c *	Electron transport	2KN9 (2009)
	Rv1739c	Sulfate transporter	2KLN (2009)
Transcription-related proteins	MT3852	ArsR family, transcription regulator, nickel metal sensor	2LKP (2012)
	Rv1994c	ArsR family, transcription regulator, cadmium metal sensor	2JSC (2013)
	Rv0639 *	Transcription elongation–termination factor	2MI6 (2013)
	Rv2050 *	RNA polymerase binding protein	2M4V (2013)
Nucleotide-binding proteins	J113_05350	DNA-binding response regulator	2RV8 (2015)
	Rv3597c *	Nucleoid-associated protein	2KNG (2009)
Ser/Thr Protein kinase-related proteins	Rv0014c *	Ser/Thr protein kinase (STPK) PknB	2KUD, 2KUE, 2KUF, 2KUI (2010)
	Rv1827	Substrate of STPK PknB	2KFU (2009)
	Rv0020c	Substrate of STPK PknB	2LC0, 2LC1 (2011)
	Rv2175c	Substrate of STPK PknL	2KFS (2009)
	Rv2234 *	Protein-tyrosine Phosphatase	2LUO (2012)
Enzymes and related proteins	Rv0733 *	Transferase, adenylate kinase	1P4S (2003)
	Rv1009	Hydrolase, Resuscitation-promoting factor	1XSF (2004)
	Rv1884c	Hydrolase, Resuscitation-promoting factor	2N5Z (2015)
	Rv1014c *	Hydrolase, peptidyl-tRNA hydrolase	2JRC (2007)
	Rv2737c	Hydrolase, endonuclease	2L8L (2011)
	MT1859	Hydrolase	2LQJ (2012)
	Rv3914	Thioredoxin	2L59, 2L4Q (2010)
	Rv3198.1	Mycothiol-dependent reductase	2LQQ, 2LQO (2012)
Siderophore-related proteins	Rv2377c *	Siderophore biosynthesis	2KHR (2009)
	Rv0451c	Siderophore export	2LW3 (2012)
Secreted proteins	Rv2875	Immunogenic protein MPT70	1NYO (2003)
	Rv1980c	Immunogenic protein MPT64	2HHI (2006)
	Rv3875/Mb3904	Type VII secretion system protein	1WA8 (2004)
	Rv0287/Rv0288	Type VII secretion system protein	2KG7 (2009)
	Rv0603	Immune system	2KGY (2009)
			2LRA (2012)
Membrane proteins	Rv1761c	Membrane protein	2K3M (2008)
	Rv0899	Pore-forming outer membrane protein	2KGS, 2KGW (2009)
			2KSM (2010)
			2L26 (2010)
			2LBT, 2LCA (2011)
Uncharacterized proteins	Rv2302	Unknown function	2A7Y (2005)
	Rv0543c	Domain of Unknown Function DUF3349 (PF11829)	2KVC (2010)
Other proteins	Rv3418c	Chaperone	1P82, 1P83 (2003)
	Rv1311	ATP synthase epsilon chain	2LX5 (2012)
	Rv0431	Vesiculogenesis and immune response regulation	2M5Y (2013)
	Rv3682 *	Penicillin binding protein	2MGV (2013)
	Rv1466	[Fe-S] cluster assembly related protein	5IRD (2016)
	Rv2171	Lipoprotein	2NC8 (2016)

* Proteins, analyzed as potential drug targets for *M. tuberculosis* in this paper.
